# Revisiting Defect-Induced Light Field Enhancement in Optical Thin Films

**DOI:** 10.3390/mi13060911

**Published:** 2022-06-09

**Authors:** Xiulan Ling, Xin Chen, Xiaofeng Liu

**Affiliations:** 1 School of Information and Communication Engineering, North University of China, Taiyuan 030051, China; 15635530642@163.com; 2 Key Laboratory of Material Science and Technology for High Power Lasers, Shanghai Institute of Optics and Fine Mechanics, Shanghai 201800, China; liuxiaofeng@siom.ac.cn

**Keywords:** optical thin film, defect, light field enhancement, laser-induced damage

## Abstract

Based on a finite-difference time-domain method, we revisited the light field intensification in optical films due to defects with different geometries. It was found that defect can induce the local light intensification in optical films and the spherical defects resulted in the highest light intensification among the defect types investigated. Light intensification can increase with defect diameter and the relative refractive index between the defect and the film layer. The shallow defects tended to have the highest light intensification. Finally, the extinction coefficient of the defect had a significant effect on light intensification. Our investigations revealed that the light field intensification induced by a nano-defect is mainly attributed to the interference enhancement of incident light and diffracted or reflected light by defects when the size of the defect is in the subwavelength range.

## 1. Introduction

Optical thin films with high laser-induced damage thresholds are the pivotal components in high-power laser systems. The laser-induced damage threshold of optical thin film affects the output energy and service life of the whole laser system. A lot of studies have shown that nano-defects caused by the fabrication of optical thin films are deemed as a potential source affecting the laser damage susceptibility [1,2,3,4,5,6]. It is believed that the laser-induced damage is the coupling damage process of multi-physical fields. Additionally, defects-induced local light field intensification is the primary, first process and the premise of subsequent thermal–mechanical damage process [7,8,9,10]. Therefore, analyzing the light field distribution of defect-induced optical films helps to understand the laser damage mechanism and develop technology to improve the anti-laser damage ability of optical films.

It has been demonstrated that defects with the spherical and nodule shapes tend to act as a micro-lens to focus the incident light [11,12], which can lead to a light intensification. However, some non-lenticular defects like surface cracks [13] also caused the local light field redistribution and enhancement. So, the mechanism of defect-induced light field enhancement in optical films is not completely attributed to the micro-lens effects.

In this paper, based on the COMSOL software, a finite-difference time-domain algorithm (FDTD), the local light field enhancement was analyzed due to the nano-defect with different geometries in SiO_2_ thin film. We also investigated light field enhancement effects induced by defects with different characteristics. Light field modeling is aimed to offer some insights into the mechanism of defect-induced light field enhancement in optical films.

## 2. Theory and Model

**Figure 1:**
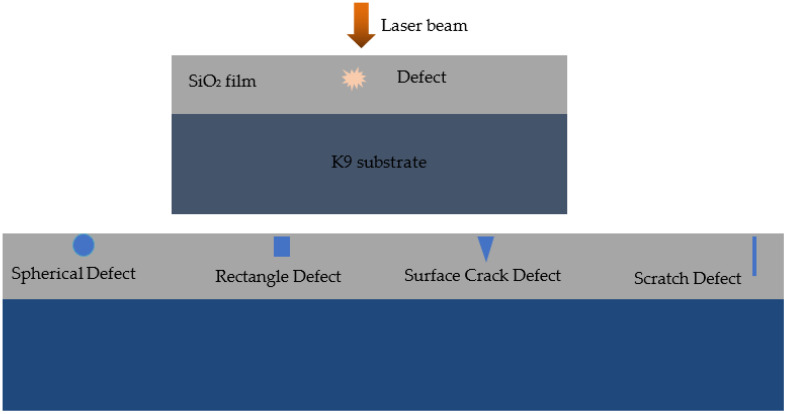
Schematic diagram of SiO_2_ film containing defects with different geometries irradiated by a laser.

We considered defects with different geometries in the single-layer SiO_2_ thin-film located on k9 substrate (borosilicate glass). The model is shown in Figure 1. As the light is a specific kind of electromagnetic wave, the propagation of laser can be regarded as the irradiation process of an electromagnetic wave. The light field intensity can be solved according to the rigorous electromagnetic field theory. The nonhomogeneous vector wave equation of the electric field can be expressed by [14]:

(1) $$\nabla \times \bar{\left(\nabla \times E\right)}-k^{2}\varepsilon_{r}E=0,$$

where ∇ is the differential operator, *E*, *k*, and $\varepsilon_{r}$ denote electric field intensity, wave number, and relative dielectric constant, respectively.

A Gaussian profile laser beam with a wavelength of 1064 nm was adopted as the incident laser and its peak electric field intensity was normalized as 1 V/m. The laser incident direction was set to be parallel to the *z*-axis, as shown in Figure 1. Thus, the component (*E_y_*) of Equation (1) in the *y*-axis direction can be described as:

(2) $$\frac{\partial }{\partial x}\left(\frac{1}{\mu_{r}}\frac{\partial E_{y}}{\partial x}\right)+\frac{\partial }{\partial z}\left(\frac{1}{\mu_{r}}\frac{\partial E_{y}}{\partial z}\right)+k_{0}^{2}\varepsilon_{r}E_{y}=0,$$

where *k*_0_ stands for the free-space wavenumber and can be described as $k_{0}=\omega \sqrt{\varepsilon_{0}\mu_{0}}$.

The simulation area was a two-dimensional region and a perfectly matched layer (PML) was adopted as the boundary condition at the bottom border, and the periodic boundary conditions were applied in the left and right boundaries. Using this model, we simulated the light field distribution induced by defects with four different shapes such as spherical defect, rectangle defect, surface crack defect, and scratch defect in mono-layer SiO_2_ film with the thickness of 300 nm and the refractive index of 1.5 [15].

## 3. Results and Discussions

### 3.1. Light Field Enhancement Induced by Defect with Different Geometries

**Figure 2:**
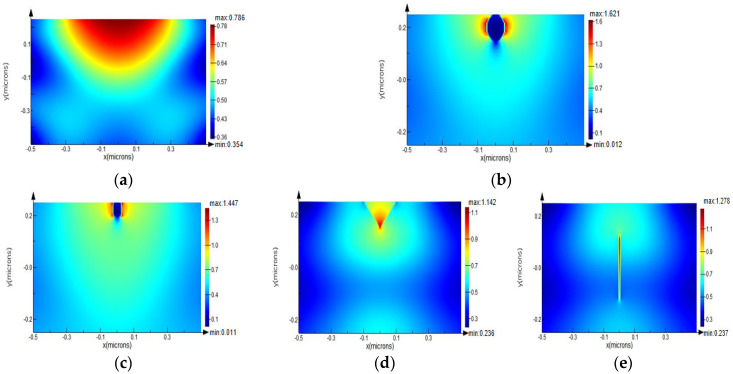
Light field enhancement induced by defects with different shapes. (**a**) without defect; (**b**) with spherical defect; (**c**) with rectangle defect; (**d**) with crack defect; and (**e**) with scratch defect.

It is observed from Figure 2 that the peak light field was located on the surface of film layer for a non-defective ideal thin-film, whereas the maximum light field lodged on the boundary of the defect for a defective thin-film. Defects not only caused a local increase in the light field intensity around the defect, also distorted the light field distribution of optical film in contrast to an ideal thin-film. Defects with different shapes induced different local light field intensifications (see Figure 2b–e), and the spherical defect induced the most significant enhancement of light field intensity. Moreover, light field intensification as large as 2.3× in the SiO_2_ thin film with the spherical defect occurred, in contrast to the condition of absence of defect. This enlightens us to reduce spherical defects such as nodule defects in optical films as much as possible.

### 3.2. Light Field Enhancement Induced by Defect with Different Characteristics 

Further analyses have been carried out to investigate the effect of defect parameters on light field intensification. Because they cause the most significant enhancement of the light field intensity, spherical defects were mainly investigated in our studies.

**Figure 3:**
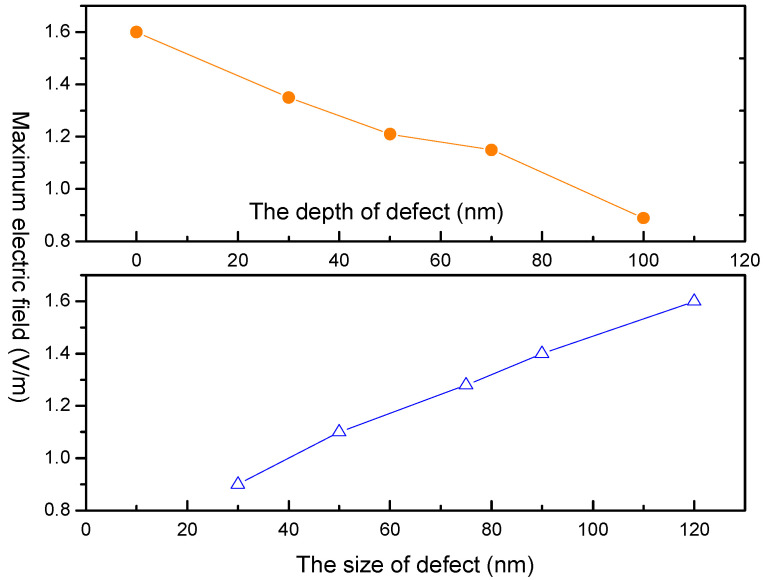
Light field intensification as a function of geometry parameters.

Figure 3 reveals that the local light field intensification induced by defects in optical film depends on the diameter and the embedded depth of the defect. It was found that with larger defect diameters, and with shallower defect embedded depths from the film layer surface, the peak light field intensifications were more significant. This result is consistent with a previous study [12]. 

**Figure 4:**
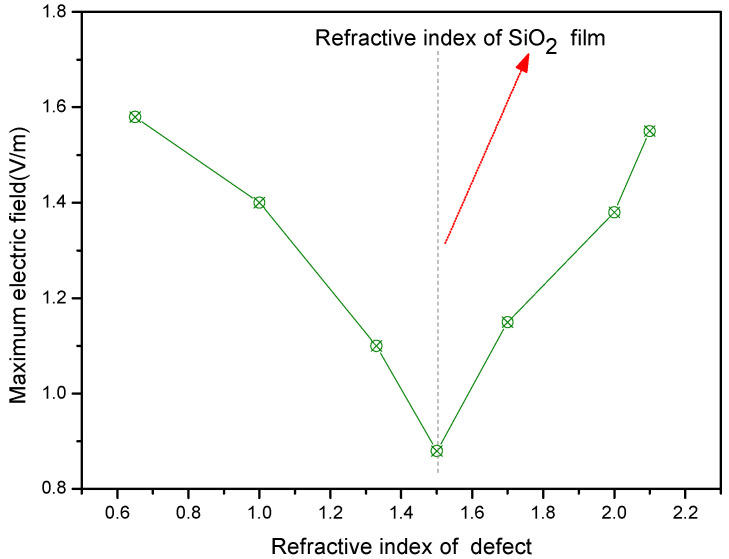
Light field intensification as a function of refractive index of the defect.

**Figure 5:**
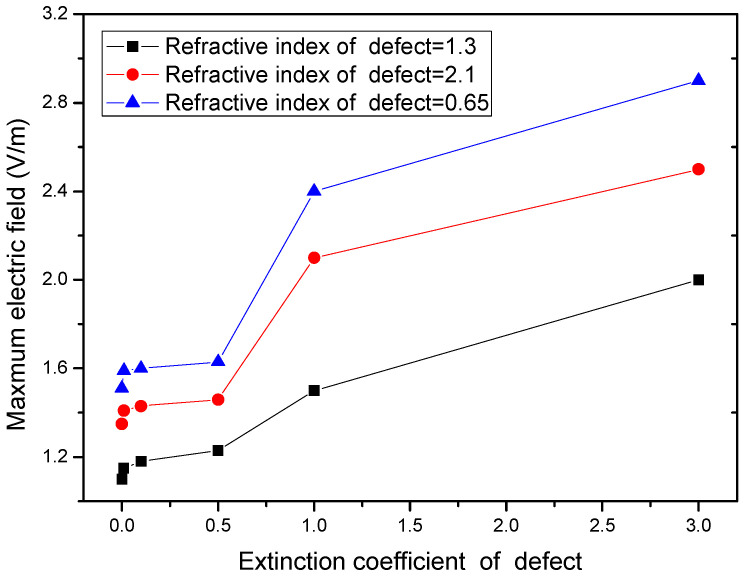
Light field intensification as a function of extinction coefficient of the defect.

In addition, the local light field intensification induced by defects is also closely related to the relative refractive index between the defect and the film layer. Figure 4 shows that the higher relative refractive index between the defect and the film layer induced a higher peak light field intensification, where the defect was located in the film and lodged at a depth of 50 nm from the film layer surface. At the same time, the local light field intensification induced by the defect also depended on the extinction coefficient of the defect. Additionally, the higher the extinction coefficient of the defect, the stronger the light field intensifications were, as shown in Figure 5. 

The previous results indicated that the defect in the film was equivalent to a micro-lens [15,16], which focused on light lead to the local light field intensification. 

Here, we revisited the micro-lens model for describing the focusing characteristics of defects. It is believed that the focusing effect of lens determined by the its focal length *f*, and that shorter focal lengths of the lens can lead to stronger focusing effects on the light. According to the formula:

(3) $$f=\frac{n}{n-1}r,$$

where *f* is the focal length, *n* is the refractive index of the medium, and the *r* is the radius of curvature of the lens, the higher refractive indexes and bigger radii of the defect lead to greater focal lengths and a weaker focusing effect on light. Moreover, according to geometric optics, the focusing effect of the lens on light should induce the light field enhancement in the incident light direction. However, the results simulated by FDTD were not in accordance to the micro-lens effect of defect. As the diameter of the defect or the relative refractive index between the defect and the coating material became higher, the focusing effect of defects on the light was more significant. So, we think that the wave property of the light plays a more important role in determining the light field enhancement effect of defects. The enhancement of light field is attributed to the interference enhancement of incident light and diffracted or reflected light by defects when the size of the defect is in the subwavelength range [17,18]. The larger defect size, the larger the relative refractive index, resulting in the stronger reflected or diffracted light, so more significant light field enhancement is induced. As the defect lodging depth increases, the reflected or diffracted light decreases, so the light field enhancement decreases, as shown in Figure 2.

**Figure 6:**
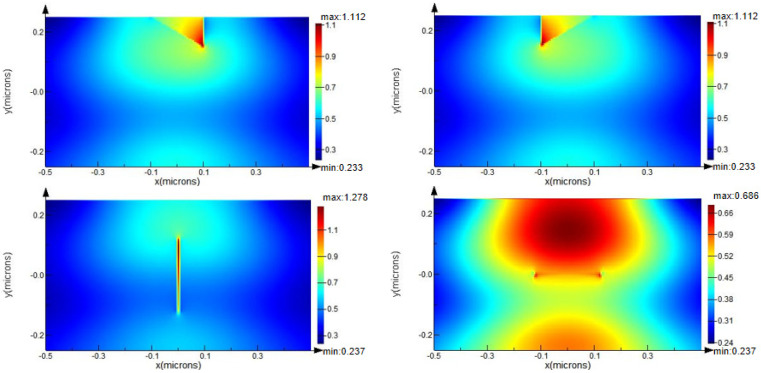
Light field distribution induced by crack defects (top) and scratch defects (bottom).

For further investigations, we simulated the light field distribution induced by two crack defects and scratch defects. Two crack defects were inclined to the propagation direction of incident light, and two scratch defects were parallel and perpendicular to the direction of incident light, respectively. The results shown in Figure 6 reveal that defects caused the reflection or diffraction of light, which interfered with the incident light and led to the light field enhancement.

### 3.3. Thermal-Mechanical Effect Due to Light Field Intensification

As is well-known, the laser-induced damage is a coupling damage process of multiple physical fields. Because the electric field intensity has a functional relationship with light intensity, the local light intensity induced by various defects is also enhanced from the results mentioned above. Absorptive defects not only regulate the light field distribution, induce the light field enhancement (see Figure 5), and lead to the local increase of light intensity *I* around defect, but also cause the increase of deposited laser energy *Q* around the defect accordingly. This can be expressed as:

(4) I=12εμ|E|2Q=∬αIdS,

where *I* is the light intensity and *E* is the electric field, *ε* and *µ* are the dielectric constant and magnetic permeability of the film layer, and α is the absorption coefficient of the defect. 

Therefore, the light field intensification results in the increasing of deposition energy around the absorbing nano-defect, which then generates a considerable temperature rise and thermal stress leading to thermal–mechanical damage [19,20]. The temperature rise and thermal stress induced by light field intensification around the absorbing nano-defects with spherical geometry and the size of 50 nm were calculated using the thermal–mechanical model [21]. It was assumed that the laser energy density corresponding to the unit electric field intensity is 10 J/cm^2^ and the absorption coefficient α was set to 0.1. 

**Figure 7:**
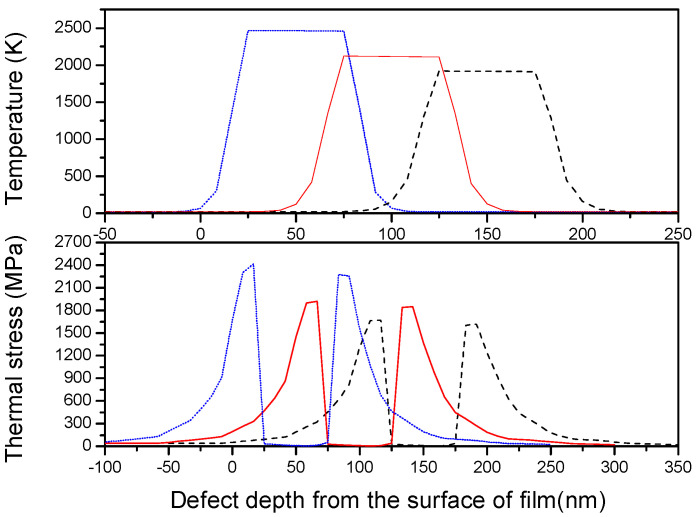
Temperature rise and thermal stress around a defect with the depth from the surface of film of 50 nm (blue line), 100 nm (red line), and 150 nm (black line), respectively.

Figure 7 shows the temperature rise and the corresponding thermal stress at the defect site with depth from the film’s surface of 50 nm, 100 nm, and 150 nm, respectively. It was found that the light field intensification induces the high temperature rise and thermal stress around defect, and the highest temperature rise and thermal stress were located in the boundary between the defect and film layer. The shallow absorptive defect lodging in the surface of film layer led to the higher temperature rise and thermal stress. This is in accordance with the conclusion that surface or subsurface defects are more likely to cause film damage [22]. Therefore, absorptive defects induce more serious laser damage of optical films through light field enhancement and the thermal–mechanical effect. 

## 4. Conclusions

We utilized COMSOL software, an FDTD code, to simulate the defect-induced light field intensification in optical films. The results showed that different geometries of defects exhibited different light field intensifications and distributions, and spherical defects induced the highest light intensification. In addition, light intensification increased with defect diameter and the relative refractive index between the defect and the film layer. The shallow defects tended to have the highest light intensification. Finally, the extinction coefficient of the defect had a significant effect on light intensification. This study also reveal that the defect-induced light field intensification is more attributed to the interference enhancement of incident light and diffracted or reflected light by defects when the size of the defect is in the subwavelength range.

## Data Availability

The data that supports the findings of this study are available within the article.
